# Selenium-based nanomaterials for biosensing applications

**DOI:** 10.1039/d2ma00756h

**Published:** 2022-09-14

**Authors:** Ebrahim Mostafavi, David Medina-Cruz, Linh B. Truong, Ajeet Kaushik, Siavash Iravani

**Affiliations:** Stanford Cardiovascular Institute, Stanford University School of Medicine Stanford CA 94305 USA ebimsv@stanford.edu ebi.mostafavi@gmail.com; Department of Medicine, Stanford University School of Medicine Stanford CA 94305 USA; Chemical Engineering Department, Northeastern University Boston MA 02115 USA; NanoBioTech Laboratory, Department of Environmental Engineering, Florida Polytechnic University Lakeland FL-33805 USA akaushik@floridapoly.edu; School of Engineering, University of Petroleum and Energy Studies (UPES) Dehradun Uttarakhand India; Faculty of Pharmacy and Pharmaceutical Sciences, Isfahan University of Medical Sciences Isfahan Iran siavashira@gmail.com

## Abstract

The unique chemical and physical features of nanomaterials make them ideal for developing new and better sensing devices, particularly biosensors. Various types of nanoparticles, including metal, oxide, and semiconductor nanostructures, have been utilized to manufacture biosensors, and each kind of nanoparticle plays a unique role in the sensing system. Nanoparticles provide critical roles such as immobilizing biomolecules, catalyzing electrochemical processes, enhancing electron transport between electrode surfaces and proteins, identifying biomolecules, and even functioning as the reactant for the catalytic reaction. Among all the potential nanosystems to be used in biosensors, selenium nanoparticle (SeNP) features have sparked a growing interest in their use in bridging biological recognition events and signal transduction, as well as in developing biosensing devices with novel applications for identification, quantification, and study of different analytes of biological relevance. The optical, physical, and chemical characteristics of differently shaped SeNPs opened up a world of possibilities for developing biosensors of biomedical interest. The outstanding biocompatibility, conductivity, catalytic characteristics, high surface-to-volume ratio, and high density of SeNPs have enabled their widespread use in developing electrochemical biosensors with superior analytical performance compared to other designs of biosensors. This review summarizes recent and ongoing advances, current challenges, and future research perspectives on real-world applications of Se-based nanobiosensors to detect biologically relevant analytes such as hydrogen peroxide, heavy metals, or glucose. Due to the superior properties and multifunctionality of Se-NPs biosensors, these structures can open up considerable new horizons in the future of healthcare and medicine.

## Abstract 2

The unique chemical and physical features of selenium nanomaterials make them ideal for developing new and better sensing devices, particularly biosensors.

## Graphical abstract

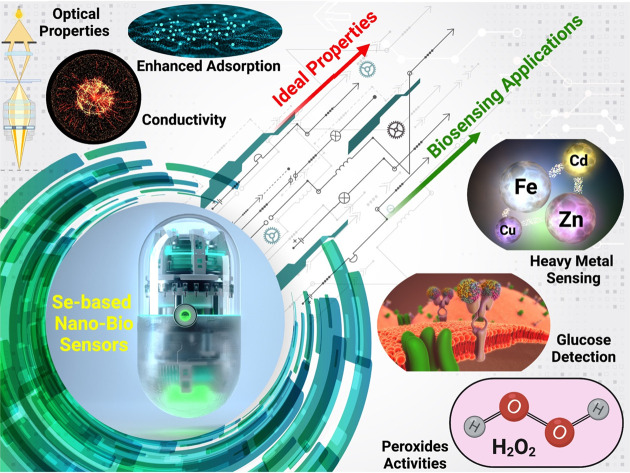

## 1. Introduction

Biological analytes such as biomolecules, biological structures, and microorganisms can be detected or quantified using biosensors as electronic devices typically composed of three components, including a device for identification of analytes and generation of signals, a signal transducer, and a reading device.^1^ Once a biosensor is assembled, the possibilities are as endless as the types of signals that can be measured. This is why biosensing has played a crucial role in controlling and diagnosing diseases, industrial product qualities, and environmental health. Continuously, there is a demand to quantify all analytes of interest in a more sensitive, accurate, and specific manner, resulting in numerous innovations in biosensors materials and designs.^2–7^

Biosensors are continually feeding on advancements in adjacent disciplines such as physics, chemistry, or nanotechnology, as any versatile and useful technology.^8^ The incorporation of metallic nanoparticles provides a novel approach to enhance the sensitivity/specificity of existing sensors.^9,10^ Various nanomaterials enable the sensing component in biosensors to be miniaturized, ensuring the commercial viability of portable biosensor kits. Additionally, surface engineering can help to develop biosensors by assuring a reproducible and stable sensing surface, analyte bio-recognition, efficient element interaction, and decreased fouling effect in a biological fluid.^11^

Among all the potential nanostructures, metallic nanoparticles (NPs) either in elemental or oxide form, exhibit the greatest potentials.^12^ Metal NPs can enhance the biosensing either through serving as the reporting molecules or as the surface at which analytes can bind and be immobilized.^13^ Several metallic NPs exhibited excellent photo-optical properties, including their widely explored localized surface plasmon resonance (LSPR), which refers to the ability for a molecule to be excited by electromagnetic wavelengths and subsequently produce intense absorption and scattering. Such plasmonic properties enhance signal generation *via* the metal-enhanced fluorescent and the surface-enhanced Raman scattering effect.^14^ By enhancing the plasmonic characteristics and refractive index of LSPR-based biosensors with metal NPs, it is possible to greatly enhance the diagnosis and monitoring of molecular markers in various diseases.^15,16^

Metallic NPs are perfect candidates for reporting molecules in place of other dyes or organic fluorophores.^17^ On the other hand, biosensors can use these inherent characteristics to interact with analytes of interest, with each metal element exhibiting unique physicochemical properties. For instance, some metallic NPs exhibit excellent electrochemical, catalytic, and conductive properties, making them suitable for electrical sensing applications.^18–21^ Additionally, an advantage of the incorporation of these materials includes their natural high surface area at the nanoscale. With increased surface area, electrodes coated with nanomaterial can immobilize and interact with more targeted analytes, resulting in higher transduced signals and, subsequently, a more sensitive assay.^22,23^

**Fig. 1:**
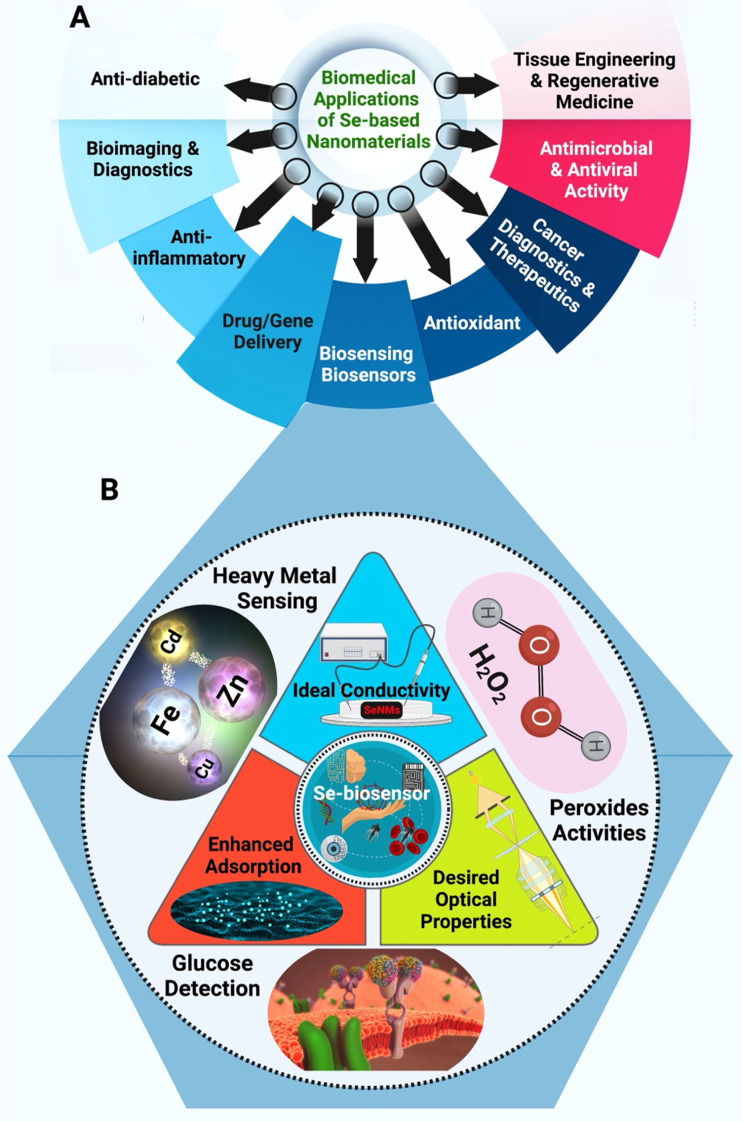
(A) Different biomedical potentials of Se-based nanomaterials (SeNMs) and (B) unique features of SeNMs that makes them promising candidates for biosensing and biosensor applications (the triangle), along with different biosensing applications that Se-based nanobiosensors can be deployed in (outer circle).

More researchers have explored metallic NPs in designing their biosensors with numerous potential benefits. To this date, metal NPs have been used to detect small molecules, oxidative agents, proteins, nucleic acid, and even microorganisms.^24–27^ It represents an incredibly exciting field, with more complex and diverse designs every day. With that said, most applications still revolve around gold and silver NPs, with copper and platinum as popular approaches.^28–31^ While these designs have proven to be successful, numerous other elemental NPs are in existence and remain unexplored.^32^ As the demand for higher quality biosensors for a wider range of target increase, some solutions may lie in this unexplored space, as each element can have uniquely beneficial properties. Selenium (Se) with anticancer, antioxidant, human immunity enhancement, and protein synthesis regulation can be applied as attractive semiconductor component in electrochemical sensors, biological sensing, solar cells, and biocatalysis/electrocatalysis.^33,34^Fig. 1A shows the most frequent bioapplications of Se-based nanomaterials. Se-based nanomaterials (*e.g.*, nanoSe, graphene-Se nanocomposites, and Se-based quantum dots) with large specific surface area, good biocompatibility, and easy of modification have been explored in designing various biosensors.^35,36^ Herein, the potential and current state of biosensors development using Se and Se-based nanomaterials are deliberated. This review will outline why Se and Se-based nanomaterials can be used to enhance biosensing applications and describe the current state of research in the field. We hope to guide and foster other research in the direction of novel elemental nanomaterials with biosensing and biomedical applications.

## 2. Selenium properties and their potential in enhanced biosensing applications

**Fig. 2:**
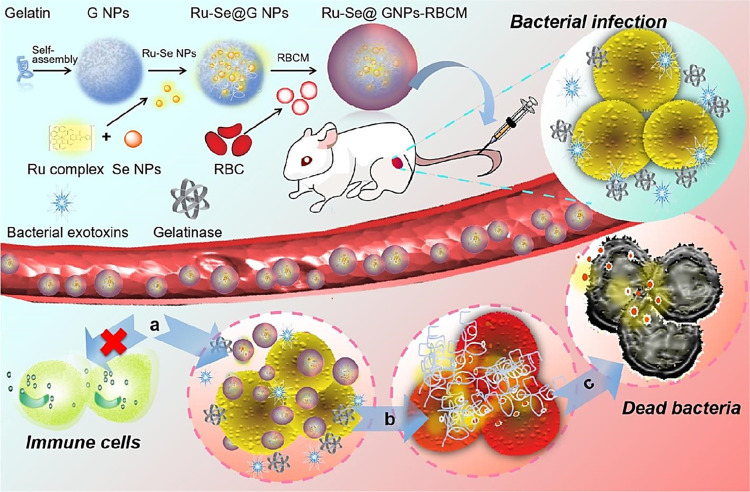
The preparative process of Se-based nanosystem for specific detection and inhibition of methicillin-resistant *S. aureus* (MRSA). RBCM: red blood cell membrane; GNP: bacteria-responsive gelatin NPs; G NPs: gelatin nanoparticles. Reproduced with permission from ref. 39.

The Se element is a nonmetal that exists at trace concentration in humans and most living organisms systems. It exhibits several desirable properties (Fig. 1A) that are of interest to numerous applications, especially in electronics and biomedical fields.^37,38^ For instance, Se-based nanosystem was constructed using natural red blood cell membrane and bacteria-responsive gelatin NPs for specific detection and elimination of methicillin-resistant *Staphylococcus aureus* based on the reactions to bacteria-infected microenvironment (Fig. 2).^39^ The application of red blood cell membrane in this nanosystem reduced the possible reactions happened by immune system, and also exotoxins were efficiently neutralized. The Se-based nanosystems with intense fluorescence imaging potentials could be applied for specific monitoring the bacterial infection treatment.^39^ However, the applications of Se and Se-based nanomaterials in biosensors have unjustly lacked behind other elemental nanomaterials such as silver or gold NPs. Nevertheless, SeNPs possess extraordinary physical and chemical properties suitable for various applications to detect biological analytes. As a semiconductor, Se shows good electrical conductivity, high photovoltaic and photoconductive properties, thermoelectric response, and exceptional oxidative characteristics.^40–42^ Besides, Se is widely used in electronics, and its application ranging from photocells to energy storage.^43^

**Fig. 3:**
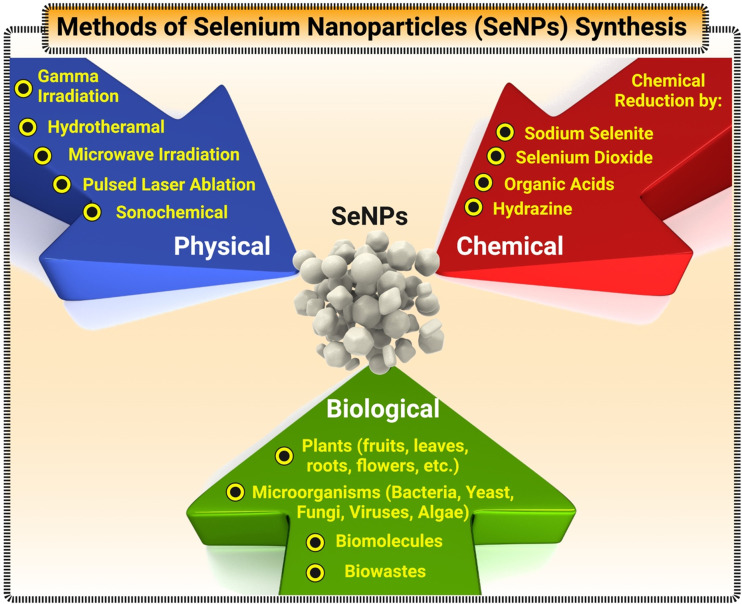
A detailed flowchart of various techniques for synthesis of SeNPs.

While bulk Se and Se ions exist in trace amounts and play an important role in regulating many cellular processes in humans, they can be toxic if surpassing a higher threshold of intake, which is approximately set at 400 μg per day.^44^ However, it has been suggested that SeNPs generated lower toxicity in biological systems.^45^ Its antimicrobial and anticancer therapy usage has been further explored, with some promising results – especially with biogenic SeNPs.^46–48^ Therefore, SeNPs have shown different properties, usually with great benefit in several use cases.^49^ Specifically, the size and morphology of SeNPs can dictate the surface-area-to-volume ratio. A higher ratio between the surface area to volume can increase the rate of adsorption, desorption, and catalysis.^50,51^ Additionally, a significant characteristic of SeNPs involves their optical properties (specifically their surface plasmon resonance) along with their regular fluorescent and tunable photoluminescence.^52,53^ Clearly, by combining the inherent properties of bulk Se with additional benefits that nanotechnology can bring, SeNPs possess an array of capabilities to (a) conduct electricity, heat, and light effects, (b) present superior surface to promote adsorptions and catalysis of interested analytes, and (c) act as the reporting molecules that generate recorded signals. Coupled with their enhanced biocompatibility, SeNPs can become a great tool in designing biosensors for detection of multiple target modalities.^32,54,55^ Currently, SeNPs can be synthesized through either physical, chemical, or biological pathways. Fig. 3 illustrates a comprehensive view of the methods utilized for synthesis of SeNPs. Physical synthesis approaches, such as laser ablation, rely on top-down formation of SeNPs from bulk materials.^56^ In contrast, chemical synthesis represents a bottom-up approach to SeNPs – reducing Se salt solution into elemental selenium.^39^ This method is usually not the favorable approach in biomedical applications due to the toxic nature of the chemicals and by-products in the synthesis process. Lastly, biogenic production of SeNPs offer a more novel and sustainable route of synthesis – utilizing existing biological machinery to produce nanomaterials.^57,58^

Additionally, researchers have finely tuned the properties of SeNPs-based sensors to expand the array of applications; for instance, the laser induced tuning of SeNPs surface plasmon resonance (SPR) peak position has been used to enhance the plasmonic application of Se quantum dots with a wide range of available light sources for resonance;^59^ while other approaches have looked into the reduction of the spatial dimension of the confinement of such nanomaterials in a precise crystallographic directions.^32,55,60^ Many researchers have made extensive attempts to improve the physicochemical properties of Se-based nanomaterials by doing strategy in order to enhance the electrochemical activity of these materials for better biosensing versatility. Urbanova *et al.* investigated the effect of Rhenium doping on enhancing the photoelectrochemical activity of Se-based materials (MoSe_2_ and WSe_2_).^61^ In another effort, Tellurium@Selenium core-shell hetero-junction was fabricated by epitaxial growth Se on Te nanotubes (NTs), which demonstrated strong broadband linear and nonlinear optical response compared to pristine Te-NTs.^62^ Other scholars comprehensively investigated the biosensing application of 2D layered materials and the impact of doping on biosensing functionality of these materials.^63–65^ The enhancement of physicochemical properties of Se have been also extensively explored using its alloying with other compounds such as Te.^66^ Functionalization of SeNPs can also transform their bandgap and make them promising candidates for optoelectronic devices.^55^ All these efforts have shown that the tunability of Se-based nanomaterials can easily overcome some of the limitations present in other biosensors based on different metals.^67–69^ Currently, the state of SeNPs-based biosensors mainly focuses on quantitating peroxides, heavy metals, and glucose – all of which will be discussed in the following section.

## 3. Current development of selenium nanomaterials in biosensing research

### 3.1. SeNPs-based nanobiosensors for peroxide detection

Various Se-based fluorescent probes were designed for specific detection of peroxides and reactive oxygen species (ROS).^38^ Peroxides, having the structure ROOR, where *R* is an element, are relevant in biology and biochemistry. When considering nanobiosensors, peroxides emerge as a critical molecule and an interesting focus for investigation. The most well-known peroxide is probably hydrogen peroxide (H_2_O_2_).^70^ This molecule is produced as a byproduct of metabolic reactions in animals, including humans. Thus, H_2_O_2_ is hazardous to human cells, owing to the peroxide ions oxidation of proteins, membrane lipids, and DNA. To combat its presence, evolution endowed cells with a family of biological enzymes known as superoxide dismutase (SOD), which is now found in virtually all live cells and functions as a key antioxidant agent.^71^ They stimulate superoxide disproportionation into oxygen and hydrogen peroxide, which promptly degrade into oxygen and water by the enzyme catalase.^72^

Without a doubt, and since this molecule is toxic to biological systems, its detection becomes critical in creating nanobiosensors. As a result, several groups have developed different generations of nanobiosensors for peroxides based on horseradish peroxidase (HRP). This enzyme catalyzes the conversion of chromogenic/chemiluminescent substrates to detect biomolecules such as proteins or nucleic acids.^73^ Some of these examples include the use of 20 to 60 nm ferromagnetic bismuth ferrite NPs modified into carbon paste electrodes with a limit of detection (LOD) at 7.0×10^−8^ M;^74^ or the development of electro-polymerized polypyrrole functionalized with platinum–palladium NPs that offered a detection range of 2.5–8000 μM along an extremely high sensitivity.^75^

**Fig. 4:**
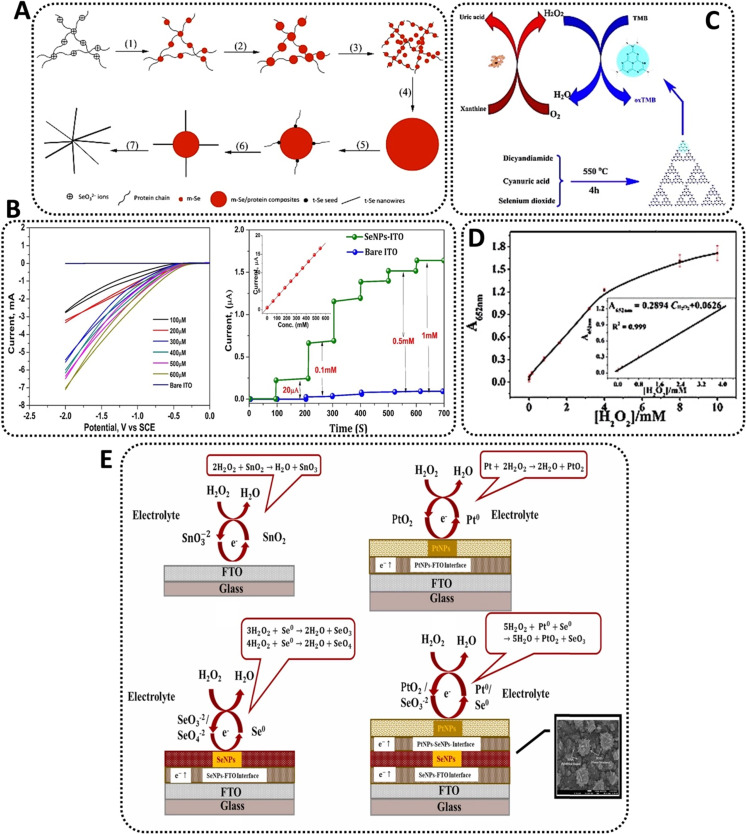
Examples of Se-based nanosensors in the detection of hydrogen peroxide; (A) schematics of monoclinic Se (m-Se)/protein composites and subsequent formation of Se-protein-nanowires^76^ (B) CVs of SeNP electrode at different Se concentrations without the presence of peroxide (left) and at different concentrations of peroxide (right);^84^ (C) schematic of colorimetric detection of peroxide and xanthine using Se-nanosheets^94^ and (D) dose-response curve for peroxide detection using Se-nanosheets.^94^ (E) The comparison between the possible mechanism of H_2_O_2_ detection on the surface of bare FTO and nanocomposites electrochemical sensors containing SeNPs.^93^

Aside from the examples above, Se becomes advantageous for detecting peroxides due to its photoelectric and semiconductor qualities, so it may be employed for this purpose, which is why some groups have begun designing biosensing platforms with SeNPs as their primary raw material. For instance, Wang *et al.*^76^ pioneered and popularized an eco-friendly biological approach to produce crystalline monoclinic SeNPs (also referred to as m-SeNPs) utilizing the Gram-positive catalase-positive bacterium *Bacillus subtilis* (Fig. 4A). The microorganism had previously been examined for its ability to synthesize SeNPs by selenite reduction *via* an inducible detoxifying pathway.^77^ As a result, the scientists treated the bacteria for 48 h with the salt precursor of SeNPs, sodium selenite (Na_2_SeO_3_). The reaction mixture changed color with time, showing that selenite ions (SeO_3_^2−^) were reduced to elemental SeNPs *via* surface plasmon vibrations of the NPs.^78^ Therefore, the created SeNPs, spherical in shape, grew in size, and became more prone to aggregation over time. For example, a 24 hour reaction yielded SeNPs with a diameter of 50–150 nm, whereas 48 h allowed for the isolation of SeNPs with a diameter of 50–400 nm. As is frequently the case with NPs generated by various bacteria, XPS studies revealed the existence of tiny SeNPs capped by proteins secreted by the microorganism.^79^ These proteins play an important role in the formation of the SeNPs, as they are often involved in the reduction and capping of the nanostructures.^80^ Besides, the long-term stability of bacterially-produced SeNPs is often due to the presence of the proteins on the surface of the NPs, which helps prevent aggregation.^81^ When the reaction mixture was aged at room temperature for 12 h, a combination of m-SeNPs and Se nanowires (SeNWs) with radial symmetry resembling a starfish were produced. To evaluate the SeNPs potential as active agents in biosensors, they were applied to a glass carbon (GC) electrode and utilized to investigate their electrocatalytic activity toward H_2_O_2_. Briefly, HRP was dripped on the electrode surface, and the peak currents associated with oxide formation/reduction were measured. The electrode treated with SeNPs produced much bigger peaks than the HRP-GC electrode used as a control. These findings were encouraging, as electron transmission between HRP and electrode is sometimes challenging. However, due to the high conductivity of SeNPs, they created an optimal microenvironment for HRP electron transport. These characteristics resulted in a much lower detection limit-around 8 × 10^−8^ M- when compared to certain other results in the literature. Additionally, the electrocatalytic response of the modified electrode was extremely stable, with both the peak potential and current remaining relatively constant throughout multiple sweeps. Interestingly, the electrode changed with m-Se had a lower potential than that modified with SeNWs. The above characteristics imply that HRP adsorbed on bacterially produced SeNPs-modified electrodes can maintain an ongoing intrinsic and favorable electrocatalytic activity toward its substrates. The utilization of SeNPs in various types of H_2_O_2_ biosensors enabled the development of an excellent electrochemical detection platform.^82^

Similarly, but more recently, cell-free extracts from an isolate of *Bacillus pumilus* sp. were utilized to synthesize stable SeNPs. This Gram-positive, aerobic, spore-forming bacillus bacterium is commonly found in soil and has been previously studied for its selenite reduction capabilities.^83^ After dissolution, aqueous SeO_3_^2−^ ions were readily converted to 20–80 nm SeNPs by adding the strain's cell-free extract to a reaction mixture. Color shifts from yellowish to red were detected after 48 h, showing that biomolecules inside the extracts successfully reduced the ions. The scientists next proceeded to investigate the potential of these crystalline SeNPs as active agents in a peroxidase biosensor by fabricating a SeNP-coated indium tin oxide (ITO) electrode for electrochemical reduction of H_2_O_2_. The reduction current progressively rose, indicating a high degree of catalytic activity. Additionally, cyclic voltammetry demonstrated that the electrode reacted to variations in peroxide concentration (at operating potential 1.0 V) and attained a steady-state signal within a few seconds at higher concentrations (up to 3.5 mM)^84^ (Fig. 4B). Accordingly, the produced nanobiosensor was a continuation of prior work but with a very cost-effective and ecologically friendly approach defined by its simplicity.

Due to Se lower electronegativity than nitrogen, it has seemed worthwhile to investigate its application as a doping agent in carbon nitride materials. As a result, the chalcogenide was chosen as a dopant in carbon nitride (C_3_N_4_) to synthesize Se doped C_3_N_4_ nanosheets by a simple calcination process. The ultrathin nanosheets demonstrated greater peroxidase mimicry activity to their undoped counterparts, catalyzing the interaction of the peroxidase substrate 3,3′,5,5′-Tetramethylbenzidine (TMB) with H_2_O_2_ to create a blue coloration. TMB, as a chromogenic substrate used in staining procedures in immunohistochemistry, is also commonly used in electrochemical reactions paired with peroxides.^85^ Additionally, xanthine, a key metabolite intermediate in both the purine nucleotide and deoxynucleotide metabolisms in mammals formed during the breakdown of adenosine triphosphate (ATP), had previously developed and been utilized to fabricate a low-cost sensor for xanthine.^86^ Thus, a colorimetric technique for H_2_O_2_ coupled with subsequent xanthine detection was devised using Se-C_3_N_4_ nanosheets built around the selective catalytic oxidation of xanthine by the enzyme xanthine oxidase (XOD).^87^

By heating a homogenous combination of dicyandiamide, cyanuric acid, and selenium dioxide, Se-doped C_3_N_4_ nanosheets were produced in a semi-closed system through a calcination process. The evenly dispersed slices had a lateral dimension of about 20 nm and were constructed of randomly aligned, hexagonally shaped graphene-like layers (Fig. 4C).^88^ They also showed a consistent particle size distribution, excellent dispersion, and a thickness of around 2 nm. At room temperature, the peroxidase-like behavior of Se-C_3_N_4_ nanosheets was examined spectrophotometrically using TMB as the peroxidase substrate in the presence or absence of H_2_O_2_, revealing that the catalytic ability of the nanosheets is significantly greater than that of C_3_N_4_ under similar conditions, which was attributed to the incorporation of Se into the system. The nanosheets facilitated electron transport, hence increasing the catalytic oxidation of the substrate TMB when H_2_O_2_ is present, providing a blue color shift in an aqueous solution. In addition, the catalytic activity of Se-C_3_N_4_ was pH- and temperature-dependent, while increasing significantly in weakly acidic solutions compared to neutral or basic solutions. This indicated that the oxidation of TMB occurs easily under weakly acidic conditions and at temperatures closer to those found in the human body, as reported for other NPs-based peroxidase sensors.^88^ Therefore, the authors found out that the Se doping of carbon nitride nanosheets and the electron transfer capabilities of C_3_N_4_ may result in a faster reaction rate. As is commonly understood, doping may encourage the creation of crystal surface imperfections, hence increasing the crystal's electrical conductivity. The C_3_N_4_ doping may have altered the crystal structure, while the Se-mediated synthesis accelerates the polymerization of the dicyandiamide and cyanuric acid precursors. These properties permitted an increase in the conductivity of Se-C_3_N_4_ nanosheets, hence enhancing the catalytic oxidation of TMB in the presence of H_2_O_2_. Due to the fact that TMB absorbs on the surface of the Se-C_3_N_4_ nanosheets and transfers lone-pair electrons from the amino groups, the electron density and mobility in the nanosheets may be significantly improved. This activity accelerates the transport of electrons from nanosheets to H_2_O_2_, hence boosting the rate of TMB oxidation by H_2_O_2_. When the catalytic sites of nanosheets were saturated with TMB, the maximum rate of conversion rose. Thus, the peroxidase-like activity of nanosheets was correlated with their ability to transfer electrons between reducing substrates and H_2_O_2_.^89^ Finally, a colorimetric approach for the detection of H_2_O_2_ was devised based on the nanosheets' inherent peroxidase enzymatic activity.^90^ Since the catalytic activity of the nanosheets was proportional to the quantity of H_2_O_2_, the concentration of this molecule may be easily measured using the naked eye or a UV-Vis spectrometer. The results revealed a distinct change, indicating that H_2_O_2_ and xanthine may be identified visually with the naked eye. This detection method, which employed nanosheets as catalysts, has a lower detection threshold than previous NPs-based techniques. Additionally, the approach was extremely selective for xanthine. Due to XOD's strong affinity for xanthine, the control experiment demonstrated that the absorbance hardly increased when other biological substances such as ascorbic acid and uric acid were added. Even when the concentration of the analogs is six times that of xanthine, there is a distinct difference in absorbance between xanthine and its analogs. Thus, the colorimetric approach devised in this study demonstrated a high degree of selectivity for xanthine identification. In clinical diagnosis, the measurement of xanthine in natural blood serum and urine samples is critical. Thus, the suggested approach for determining xanthine was validated in actual samples using the nanosheets-catalyzed colorimetric detection. The analysis of buffer solution, blood, urine, and coffee samples suggested that the proposed method may be used to detect xanthine in any type of biological sample. The reproducibility of the sensor was determined by analyzing H_2_O_2_ and xanthine at 4 mM and 160 mM, respectively, finding out that after one week of storage, the biosensor retained 95.9% of its original catalytic activity, indicating that the platform exhibited good storage stability.^91^

SeNPs were coated on fluorine-doped tin oxide glass substrate *via* a spin coating technique to detect H_2_O_2_. Consequently, the prepared sensor exhibited high sensitivity for H_2_O_2_ (∼104.2 μA mM^−1^ cm^−2^); the linear detection range was ∼0.1 to 20 mM with LOD of ∼0.0793 mM.^92^ Similarly, a nanocomposite was constructed from platinum (Pt) NPs, SeNPs, and fluorine doped tin oxide (FTO) to develop an electrochemical sensor for sensitive detection of H_2_O_2_. Accordingly, the designed sensor exhibited the linear detection range of 0.01 to 40 mM and response time of 0.5 s, with sensitivity of ∼7.3 μA mM^−1^ cm^−2^.^93^ These SeNPs-based sensors can be applied for detection of H_2_O_2_ with high selectivity and sensitivity. Fig. 4E compares the proposed mechanism of H_2_O_2_ detection on the surface of bare FTO with nanocomposites electrochemical sensors containing SeNPs.^93^

### 3.2. SeNPs-based nanobiosensors for heavy metals detection

Another significant target analyte for NPs-based sensors comes from heavy metals, whose presence has grown significantly in various habitats, resulting in a huge global environmental problem. In their elemental state, ions released from heavy metals have a long half-life and can bioaccumulate within multiple trophic chains.^95^ Additionally, once these ions enter the body, they are frequently non-biodegradable, harming human health.^96^ At the moment, heavy metals are detected using a variety of standard spectroscopic and chromatographic approaches.^97^ Nonetheless, these are subject to a variety of constraints.^98^

Numerous approaches for heavy metal analysis have been adopted using nanomaterials, including electrochemical, colorimetric, fluorescence, and biosensing technology; and multiple types of NPs have been used in the removal of heavy metals, including metal oxide NPs, magnetic NPs, graphene, and its derivatives, and carbon nanotubes.^99,100^ As a result, the use of NPs may prove advantageous in creating creative techniques to circumvent these constraints.^98^ For instance, a novel DNAzyme biosensor for ultra-trace Pb^2+^ detection was developed recently based on the “hot spot” effect in the near-infrared band using AuNPs. In the system, Pb^2+^ with high catalytic activity can cleave a DNAzyme-linked ribonucleotide adenine-containing substrate strand. This process triggers the conversion of the DNA double helix structure to a single strand and causing the AuNPs bound to DNAzyme;^101^ while another group employed a bimetallic surface-enhanced Raman spectroscopic (SERS) nanobiosensor built with gold/silver-bimetallic NPs for the detection of solid waste landfill leachate in groundwater and the potential to find traces of heavy metals.^102^

Owing to their mechanical and electrochemical properties, SeNPs have been used as key components in building similar platforms for detecting heavy metals in different substrates. Recently, the selenite-reducing bacteria *Stenotrophomonas acidaminiphila* was used to synthesize SeNPs assembled to produce a biosensor to detect heavy metals in aqueous solutions. Between 16 and 72 h of incubation in the presence of the ions, Na_2_SeO_3_ concentrations up to 100 mM generated a considerable number of SeNPs. The red coloration of the bacterial supernatant was attributed to an enzymatic mechanism in which selenate is reduced to elemental Se in the presence of nicotinamide adenine dinucleotide (NADH). Taking these findings into account, the scientists decided to design an assay for detecting heavy metal toxicity based on selenite reduction, as altering the NADH reductase-dependent synthesis of SeNPs may be used to measure the presence of other ionic interferents. With this objective in mind, the unique fluorescence spectrum of NADH –a fluorophore that absorbs light in the 335–350 nm range with the emission peak around 440–470 nm^103^ –was employed to identify enzymatic activity; thus, the decrease in fluorescence intensity was associated with the differential toxicity of heavy metals. The results indicated that when the reaction mixture was exposed to a fixed concentration of different metal elements-including iron (Fe), zinc (Zn), cadmium (Cd), arsenic (As), and mercury (Hg)–, the fluorescence was quenched to a greater extent than when the reaction mixture was exposed to the control conditions. As a result, decreased fluorescence indicates the production of a less quantity of SeNPs. Therefore, it was discovered that supernatant devoid of analyte exhibited a heightened red color, which might result from increased selenate reduction in the absence of heavy metals. Optical examination of the supernatant containing hazardous heavy metals corroborated the decrease in SeO_3_^2−^, as shown by a decrease in color intensity relative to the control. Due to the reduction in optical signal, the established platform may be utilized to evaluate a range of xenobiotics. Therefore, this ecologically friendly and easy-to-use method may be useful for identifying heavy metals through a stringent toxicity evaluation.^104^

**Fig. 5:**
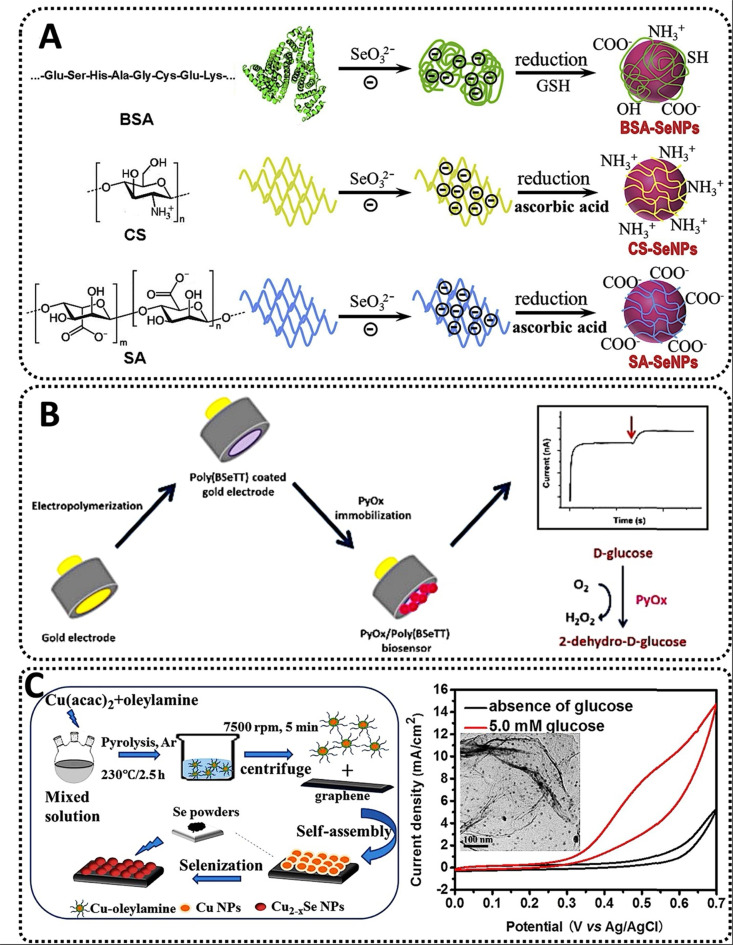
(A) The synthesis scheme for three types of SeNPs stabilized with BSA, CS and SA, respectively;^107^ (B) construction of a pyranose oxidase biosensor based on selenium-containing conducting polymer for glucose detection;^108^ and (C) the manufacture of monodisperse copper selenide (Cu2-*x*Se) nanoparticles that are generated by thermal degradation of copper acetylacetonate (Cu(acac)_2_) in the presence of oleylamine and applied to a GO support for glucose sensing.^109^

Similarly, a recent work examined the influence of stabilizers on the oxidase-like activity of SeNPs as a method to detect heavy metals. Stabilizers such as bovine serum albumin (BSA), chitosan (CS), and sodium alginate were evaluated (SA) as main raw materials. While BSA is a serum albumin protein taken from cows used as a standard for protein concentration in laboratory investigations, CS and SA are polysaccharides formed from shellfish and brown seaweed's hard outer skeletons. SeNPs were produced by reducing Na_2_SeO_3_ with glutathione or ascorbic acid in the presence of the various stabilizers. All SeNPs were spherical and had an average size of around 40 nm (BSA-SeNPs), 70 nm (CS-SeNPs), and 60 nm (SA-SeNPs). The existence of the stabilizer's specific absorption peaks in the spectra of SeNPs further highlighted its essential function in the synthesis of SeNPs. Next, the catalytic oxidation of the oxidase substrate TMB was examined to establish whether or not all SeNPs exhibited oxidase-like activity. While naked SeNPs were incapable of catalyzing the oxidation of TMB to its blue oxidized state, the three kinds of SeNPs with the stabilizer demonstrated oxidase-like activity, with CS-SeNPs doing the best. SeNPs exhibited pH- and temperature-dependent catalytic activity. Moreover, whereas both BSA- and CS-SeNPs displayed the same affinity for TMB, the catalytic constant of CS-SeNPs was about fourfold that of BSA-SeNPs. Notably, the authors recognized that while Se is an effective antidote to Hg poisoning *via* the formation of less toxic mercuric selenide (HgSe), no attempt had previously been made to build a Se-based biosensor for Hg^2+^. The results indicated that Hg^2+^ significantly inhibited the oxidase-like activity of CS-SeNPs and that they could catalyze the oxidation of TMB to generate the blue product oxidized TMB; thus, the selectivity of the system was evaluated for various metal ions-Cd^2+^, Pb^2+^, Cu^2+^, Na^+^, K^+^, Ca^2+^, Mg^2+^, Zn^2+^, and Mn^2+^-. The comparatively high selectivity and sensitivity of the Hg^2+^ detection technique can be related to the strong binding affinity between Hg2+ and Se, since chalcogen is the ideal binding partner for the heavy metal.^105^ Then, in the generated system, Hg^2+^ would bind particularly to the active Se atom on the surface of CS-SeNPs, hence limiting electron transport from TMB to oxygen and CS-SeNPs oxidase-like activity. CS-SeNPs were favorable for Hg^2+^ detection in two ways, despite the fact that the detection limit was low compared to other existing systems: (1) The nanobiosensor was considerably cheaper than sensors produced from noble metals such as Pt, Pd, or Au;^106^ and (2) CS-SeNPs possessed oxidase-like activity, which could catalyze the oxidation of TMB without the presence of unstable H_2_O_2_, whereas other enzymes used cannot achieve such performance. Thus, based on the oxidase-like activity of CS-SeNPs, a simple and economic colorimetric test for Hg^2+^ was developed (Fig. 5A).^107^

### 3.3. SeNPs-based nanobiosensors for glucose detection

Without a doubt, glucose biosensing in diagnosis and therapy is critical due to the global incidence of diabetes while also becoming increasingly important in the food and pharmaceutical sectors. Glucose sensing has historically been achieved using a variety of tactics, including electrochemical or optical approaches.^110^ However, patients may experience pain and localized infections due to the demand for repeated finger pricking to collect a blood sample for glucose sensing. Alternately, continuous glucose sensors have been developed, which follow your glucose levels continuously using a tiny sensor worn on the back of the upper arm and check glucose concentrations.^111^ However, the internal sensor must be replaced often, and the device must be routinely recalibrated using a conventional finger stick. Additionally, there is a chance of skin infection and false alerts.^112^ As a result, patient-friendly, minimally invasive, or non-invasive fluorescence detection technologies have attracted interest, and some have made significant advances when combined with nanotechnology. The majority of these nanobiosensors are enzymatic or non-enzymatic and are based on the chemisorption of highly active glucose oxidase (GOx) onto AuNPs;^113^ or by the chemical coating of different active components into carbon-based electrodes, such as high-purity TiO_2_,^114^ silver (Ag)^115^ or nickel (Ni)^116^ in the nanoscale, among others.

Indeed, the application of SeNPs in glucose sensors has not been as deeply explored as those using different noble metals. Nonetheless, some interesting examples have shown the potential of the chalcogen as a key component in the detection of glucose. For example, a selective and sensitive glucose biosensor was produced by immobilizing the oxidoreductase enzyme glucose oxidase (GOD) onto a matrix composed of SeNPs and mesoporous silica (referred to as MCM-41) that was applied to a carbon paste electrode (CPE). While MCM-41 (short for Mobil Composition of Matter No. 41) is a mesoporous material produced by the Mobil Oil Corporation and consisting of silicate and alumosilicate particles,^117^ Se-MCM-41 is produced *via* covalent bonding of SeNPs to the framework of MCM-41 by reagents such as 3-(Triethoxysilyl)-propylamine (APTES).^118^ The electrode was made by combining a glutaraldehyde-crosslinked Se-MCM-41 mesopore composite with Aspergillus niger-produced GOD. Utilizing a CPE-modified working electrode, the electrochemical behavior of the Se-MCM-41-GOD mesopore composite was analyzed. The glucose was oxidized catalytically by GOD, and immobilized GOD retained its electrocatalytic activity, indicating that GOD bound to the Se-MCM-41 composite was active in electron transfer. Electron transport may potentially be accounted for by electrostatic interactions between MCM-41 and oxidized glucose mediated by hydrogen bonding and hydrophilic attraction.^119^ Next, the authors chose to investigate the effect of pH and glucose concentration on the sensor performance. On the one hand, a pH range of 4–8 was employed, paired with a monopotassium phosphate buffer solution containing 2 mM glucose at 25 °C, demonstrating that the sensor can detect glucose levels in actual samples with a neutral pH value. On the other hand, glucose concentrations ranging from 0.01 to 12 mM were tested, resulting in a linear response range of the sensor between 0.01 and 2.0 mM. Notably, the stated sensitivity exceeded that of commercially available carbon paste sensors based on carbon nanotubes or comparable film electrodes. In addition, repeatability and stability tests demonstrated that the electrode maintained 91% of its original current response while stored at 4 °C for glucose for 10 days with occasional usage. Lastly, it is well-established that physiologically relevant doses (0.1 mM for ascorbic acid and 0.1 mM for citric acid) inhibit the sensing applicability of similar systems. However, when the glucose-to-interference concentration ratio was 1 : 1, the presence of interferences had no noticeable influence on the current system measurement of the GOD, indicating a high degree of selectivity.^120^

Similarly, an amperometric pyranose oxidase (PyOx) biosensor based on a Si-containing conducting polymer was recently developed. In comparison to GOX, PyOX has a greater power output since it is not glycosylated, is capable of catalyzing the conversion of both anomers of glucose, and interacts with a broader spectrum of substrates.^121^ Notably, PyOX does not result in an undesirable pH change. On a gold electrode, the conducting polymer poly(4,7-bis(thieno[3,2-*b*]thiophen-2-yl)benzo[*c*][1,2,5] selenadiazole) (also known as poly(BSeTT)) was produced through electropolymerisation. PyOx was then immobilized on the polymer using glutaraldehyde. The morphology of the Se-containing composite resembled a homogeneous cauliflower-like structure, however the surface morphology was considerably affected by molecule orientation following enzymatic immobilization. After adjusting the polymer characteristics to meet the specifications of the biosensor, the effect of pH on the stability was examined using 0.5 mM glucose as the substrate and 50 mM buffer solutions with pH values ranging from 4.5 to 10. Simultaneously, interferents such as ascorbic acid and urea (with concentrations ranging from 0.1 to 1 mM) were analyzed, and substrate specificity was evaluated using a range of sugars, such as xylose, fructose, galactose, mannose, and sucrose. Notably, the experiments demonstrated that using a conductive polymer containing Se improved operational stability. Conducting polymers containing Se can enhance performance, whereas Se alone acts as an antioxidant, preventing tissue cell degradation and creating a friendly environment for biosensor creation. Finally, under optimum conditions, the suggested biosensor was used to determine the glucose content in various liquids, including coke, iced tea, and milk. The obtained data were in excellent agreement with the manufacturers’ databases, demonstrating the biosensor system accuracy. The biosensor was an accurate platform for glucose testing in actual samples with the potential for real-time analysis (Fig. 5B).^108^

Rather than SeNPs alone, different combinations of Se with other metals in the nanoscale have been tested for the building of glucose biosensors. One of the most remarkable examples comes from copper selenide (CuSe) NPs, which have advantageous characteristics for a wide variety of applications. This composite nanomaterial is suitable for thermoelectric and photovoltaic energy harvesting, photocatalytic degradation of dyes for water purification, and manufacturing of battery electrodes.^122^ Furthermore, CuSe has also been investigated for use in the treatment of colon cancer using photothermal therapy.^123^ CuSeNPs adopt several different crystal structures and compositions in the nanoscale, depending on the synthesis method employed.^124^ When CuSe is used as a biosensor, different groups have examined its unique features, including its low working potential and selectivity for glucose sensing over other biomolecules found in body fluids. Additionally, CuSe has improved electrochemical performance in the oxidation of glucose, which can be due to numerous factors. Since the first step in glucose oxidation is catalyst activation, which is achieved by covalently bonding the molecule to the electrode surface *via* the –OH functional group on the catalytically active transition metal site (such as the one provided by Cu), the –OH attachment results from the active site being locally oxidized.^125^ Certain groups have showed that –OH adsorption may be accelerated by modifying the ligand environment, often by reducing the anion electronegativity, which decreases the necessary activation potential for the catalyst and boosts its efficiency. The covalency of Cu–Se links then rises in CuSe. This variety of oxidation states creates an inductive effect and redistributes the electron density at metal sites *via* d–d interactions, which favors –OH group adsorption. In addition, it is feasible to improve the lattice covalency and redox activity at the Cu site by replacing less electronegative selenides for oxides, which results in a reversible electrochemical reaction. Accounting for such features, recently, monodisperse CuSeNPs were synthesized through the thermal breakdown of copper acetylacetonate and combined at various temperatures with reduced graphene oxide (RGO), which served as a support. The physicochemical examination revealed a high concentration of carbon WHICH and oxygen (O), indicating that carbon derived from graphene and the oxygen in the air were absorbed by the CuSeNPs catalyst during the selenization process. The samples were then deposited on the prepared carbon fabric to create an electrode. When a particular quantity of glucose was introduced, the CuSeNPs had a considerably increased current response, implying that the NPs greatly boosted the catalytic activity for glucose oxidation-reduction compared to CuNPs alone. This might be explained by the selenization of CuNPs, which enhanced the electrochemically active surface area and facilitated mass diffusion during the electrocatalytic method.^126^ The biosensor selectivity was determined in the presence of various sugars, such as maltose, sucrose, mannose, and fructose, all at a concentration of 0.1 mM. Reproducibility was also excellent, with the sensor being stable and reproducible for seven days, with a 20% decline in current response relative to the original current following this period (Fig. 5C).^109^

Similarly, a CuSe thin film was directly produced on a carbon cloth electrode using a three-electrode setup consisting of an Ag/AgCl reference electrode, a graphite rod counter electrode, and commercial carbon cloth as the working electrode. CuSe has a nanoflake-like surface structure, as indicated by SEM analysis of the as-deposited CuSe thin film. The random orientation of the nanoflakes produced a porous coating with a wide surface area for glucose adsorption. The electrocatalytic performance of the CuSe thin film for the oxidation of glucose in the presence and absence of 0.1 mM glucose was investigated using cyclic voltammetry. The anodic current increased significantly when the sugar was added to the alkaline electrolyte, showing glucose oxidation on the CuSe-coated electrode. The electrochemical reaction was linked to the analyte's oxidation on the electrode surface. Further investigation revealed that the glucose oxidation peaks exhibited a clear tendency toward increasing current as of the scan rate increases and a positive change in the anodic oxidation potential. The applied potential for glucose sensing was obtained using an amperometric approach in which 0.1 mM glucose was added sequentially while swirling continuously. The optimal working potential for glucose oxidation was +0.15 V *vs.* Ag|AgCl. Furthermore, chronoamperometry was used to determine the reaction of the CuSe composite electrode and CuSeNPs powder to sequential glucose injections in a homogeneously stirred sodium hydroxide (NaOH) solution. The decreased sensitivity of the hydrothermally produced powder was attributed to the presence of Nafion in the composite electrode which prevents the catalytic site from being exposed to glucose in the electrolyte.^127^ In contrast to previous non-enzymatic glucose sensors described in the literature, the CuSe-based electrodes demonstrated a high sensitivity for glucose detection with a low LOD. To evaluate the long-term functional stability of the electrodes, glucose oxidation currents were recorded by exposing the same electrode to a 1 mM glucose solution every day for over 30 days and keeping it at room temperature. Both electrodeposited and hydrothermally synthesized CuSe-modified electrodes retained better than 90 percent of their original current responsiveness after 30 days of exposure to air. Lastly, the practical use of the non-enzymatic glucose sensor was assessed by detecting glucose concentrations in human blood samples. The blood sample was injected directly into the NaOH electrolyte near the CuSe-modified electrode, and the current response was recorded; the results were equivalent to those obtained using commercial enzyme biosensors with low error.^128^Table 1 summarizes some of the most recent studies utilizing SeNPs-based biosensors for detecting various analytics.

**Table 1:** Different examples of SeNPs-based biosensors

Detected analytics	Nanoparticle component	Nanoparticle size (nm)	Limit of detection (LOD)	Linear range of detection	Sensitivity	Ref.
H_2_O_2_	*Bacillus subtilis* produced crystalline SeNPs	50–400	8 × 10^−8^ M	0.8 × 10^−8^–10^−5^ M	Undefined	129
H_2_O_2_	*Bacillus pumilus* sp produced SeNPs	20–80	3 μM	5–600 μM	16.54 μA mM^−1^ cm^−2^	130
H_2_O_2_ Xanthanide	Se doped C_3_N_4_ nanosheets	20	1.6 × 10^−5^ (H_2_O_2_), 1.6 × 10^−8^ mol L^−1^ (Xanthanide)	1.6 × 10^−5^ to 4 × 10^−3^ mol L^−1^ (H_2_O_2_), 1.6 × 10^−^7 to 4.0 × 10^−5^ mol L^−1^ (Xanthanide)	Undefined	131
Heavy metals	*Stenotrophomonas aidaminiphila* produced SeNPs	35–40	Undefined	0.01–2 mM	Undefined	132
Heavy metals	Stabilizer-based SeNPs	40 nm (BSA-SeNPs), 70 nm (CS-SeNPs), 60 nm (SA-SeNPs)	0.12 uM	0.1 to 2.5 μM	Undefined	133
Glucose	Selenium crosslinked to MCM-41	Undefined	1 × 10^−4^ M	0.01–2 mM	0.34 A·mM^−1^	134
Glucose	Poly(BseTT) composite attached to PyOx	Undefined	3.3 × 10^−4^ nM	0.02–0.5 mM	6.4 nA mM^−1^ cm^−2^	135
Glucose	CuSeNPs combined with GO	10	0.05 mM	0.1–3.375 mM	536 μA mM^−1^ cm^−2^	136
Glucose	CuSe thin film on a carbon cloth	Undefined	196 nM	100 nM to 40 μM	19.419 mA mM^−1^ cm^−2^	128

## 4. Conclusions and future outlooks

Nanomaterials with unique physicochemical properties exhibited excellent potential for developing analytical systems with appealing and promising biosensing behaviors for specific detection, quantification, and evaluation of different biomolecules in living organisms and important toxins in the environment linked to life cycles. In this context, Se-based nanomaterials with excellent conductivity and electrochemical features can be applied as efficient electrode materials in designing sensors with high sensitivity and selectivity. Extensive research has established the favorable properties of different NPs for distinct types of biosensors. Therefore, whatever function NPs play in the biosensing process, they significantly improve the analytical performance of biosensors in terms of sensitivity, selectivity, and reliability. However, from the standpoint of practical, real-world applications, this rapidly expanding field is still in its infancy, and extensive practical deployment of NPs-based biosensors is not yet extensively feasible. Before realizing the full potential of NPs for biosensor applications, many hurdles must be overcome. Therefore, one of the main approaches that the field should take is to expand the current pipeline of available raw materials for the production of valuable NPs in biosensors.

Aside from commonly used metals, such as gold, silver, or iron composites, the employment of selenium has been somewhat studied as a possible way to overcome the limitations of pre-existing platforms and also to provide a novel array of possibilities owing to the physicochemical features of the chalcogen. Consequently, different biosensors for detecting peroxides, heavy metals, or glucose, among other analytes, had been discovered, developed, and tested in the field, showing promising results that might evolve into a novel array of SeNPs-based biosensors. Additionally, the use of novel Se nanostructures and nanoconjugates should be explored in order to further expand their applications. Bi-elemental and multi-elemental nanobiosensors can optimize the desirable properties of each starting materials, and therefore should be studied.

Nevertheless, to fully leverage the potential use of SeNPs in biosensors, attention should be paid to creating biosensors capable of high-throughput and multiplexed detection of biomarkers. Another intriguing area of research is the long-term stability of SeNPs in various settings. Additionally, because the size and nanostructure of SeNPs have a significant influence on their optical and electrical characteristics, research on the design and synthesis of NPs with well-defined geometry and attributes will boost their use in biosensors. Combining SeNPs with other nanomaterials, such as copper, is an exciting path to pursue. With the vast array of newly discovered nanomaterials, hybrids of SeNPs and other nanomaterials may exhibit different features. Along with exploring novel structures or compositions, efforts to enhance analytical performance at the current level, such as limiting nonspecific adsorption of biomolecules onto SeNPs or shortening the analysis time, require thorough consideration. Future SeNPs-based biosensing systems should also incorporate microfluidics and micro-optical components to combine sample processing and particle interrogation into a single hand-held device that enables on-site sample inspection.

## Author contributions

The idea of the review was conceived by E. M., D. M. C., and L. B. T. The original draft of the manuscript was written by D. M. C., L. B. T., and E. M. Review and editing of the manuscript was performed by E. M., S. I., and A. K. All original illustrations/figures were created by E. M. Study was supervised by E. M. All authors have read and agreed to the published version of the manuscript.

## Conflicts of interest

The authors declare no competing interests.

## Supplementary Material
